# Use of pattern-recognition display techniques to visualize the data contained in complex data-bases. A case study

**DOI:** 10.1155/S1463924683000346

**Published:** 1983

**Authors:** M. P. Derde, D. L. Massart, W. Ooghe, A. De Waele

**Affiliations:** 1 Farmaceutisch Instituut Vrije Universiteit Brussel Laarbeeklaan 103 Brussels B I090 Belgium; 2 Farmaceutisch Instituut Rijksuniversiteit Gent Harelbekestraat 72 Gent B 9000 Belgium


Journal of Automatic Chemistry, Volume 5, Number 3 (July–September 1983), pages 136–145

### Use of pattern-recognition display techniques to visualize the data contained in complex data-bases. A case study

**M. P. Derde, D. L. Massart*,**

*Farmaceutisch Instituut, Vrije Universiteit Brussel, Laarbeeklaan 103, B 1090 Brussels, Belgium*

**W. Ooghe and A. De Waele**

*Farmaceutisch Instituut, Rijksuniversiteit Gent, Harelbekestraat 72, B 9000 Gent, Belgium*

### Introduction

Automation and computer acquisition of data in analytical chemistry has meant that enormous amounts of data can be obtained. The analytical chemist is faced with the problem of bringing some order into these data, so that he can understand the relationships between the variables being measured and between those variables and the object of his research. This is particularly difficult when more than two variables are being measured. Indeed, when only one or two variables are measured the analyst usually makes graphs to explain what happens. When more than two variables are measured, it is impossible to represent the data in two dimensions. The purpose of this article is to show that, in such a case, pattern-recognition methods can be used to visualize the data and therefore to gain insight into them. These methods can be called display methods. Instead of giving a lot of theory, this is demonstrated with a practical example: the authentification of the geographical origin of wine.

The classical methods for the chemical analysis of wines enable the major constituents, such as ethanol content, dry matter, acidity and mineral content, to be controlled. These parameters can be useful for the detection of adulterations, but do not help with the problem of the identification of wines according to their origin. Until now, such an identification has only been possible with sensorial evaluation by experienced tasters.

As the characterization of wines according to origin is important, several investigators have attempted to solve the problem by multivariate characterization of wines. Wines are analysed for a number of constituents and the set of observations made on each wine sample constitutes a pattern. If the constituents that were analysed were appropriately chosen, wines from different origins would have different patterns—so these patterns may be used for classification according to origin. Moret *et al.* [1] and Scarponi *et al.* [2 and 3] analysed samples of three groups of Venetian DOC wines for several inorganic parameters. On the basis of the patterns obtained, and with the use of multivariate statistical techniques, the three groups of wines could be reasonably well identified. Kwan *et al.* [4] also proved that the characterization of wines on the basis of inorganic contents can be useful for assignment according to origin. Schreier *et al.* [5] made use of the concentrations of aroma constituents derived from the grapes: the six groups of German white wines that were involved in their study could be discriminated using multivariate techniques. When the same samples were characterized by their content of yeast metabolites, the discrimination decreased considerably.

It has been suggested that amino-acid patterns may be useful for the detection of adulterations of food products (orange-juice for example) [6, 7 and 8], and in order to determine the geographical origin of foodstuffs. Gilbert *et al.* [9] made use of amino-acid patterns to prove that groups of honey samples from different countries could be distinguished from each other.

As amino-acids are important factors in the vinification process, and as most of the amino-acids in wines are related to the grape varieties, Ooghe and De Waele [10] suggested that the differentiation of wines according to origin is possible on the basis of such patterns. Therefore, French wines were analysed for 20 amino-acids. The interpretation of the resulting data-set is not possible with the use of univariate statistics or by visual interpretation. The evaluation of the results by comparing the values for each of the parameters separately takes no account of the relationship between these different parameters. This can cause an important loss of information, as becomes obvious from the following example. In figure 1 the concentration of the proline content is plotted against the concentration of the glycine content in, respectively, 13 Côtes du Rhone wines and 14 Beaujolais wines. From the range of these two parameters in both groups it is clear that univariate consideration of the two parameters separately does not permit differentiation of both groups of wines. However, when the two parameters are plotted against each other, a good discrimination can be made between the two groups. Obviously, more information is obtained from bivariate interpretation of the results. If the concentration of a third amino-acid is taken into consideration, the data-set can be visualized by representing each individual wine sample in a three-dimensional space, each dimension corresponding to the concentration of one acid. This three-dimensional representation might then improve the differentiation between the groups. However, only a minor part of the information included in the data-set is used. As 20 parameters are available for each wine, each of the samples can be thought to be situated in a 20-dimensional space. Visual representation of the data is no longer possible—more sophisticated techniques that utilize the information in an optimal way and that can give a visual representation of the results are necessary. Multivariate statistical techniques or pattern-recognition techniques can be used for this purpose.

Among the different pattern-recognition techniques, a distinction can be made between three groups—pure display methods, supervised methods and clustering techniques:

(1) The aim of the pure display methods is to represent multivariate data collected for a group of samples in a two-dimensional space without significant loss of information.
(2) Supervised methods are used when the data-set consists of samples that can be divided *a priori* into several groups, which is the case in this example. They aim to develop mathematical decision rules that can be used for the classification of new samples.

* Professor Dr Massart is corresponding author.

(3) Clustering techniques are employed when the samples of the data-set cannot be *a priori* divided into groups, or when one ignores the *a priori* existence of groups. The fundamental purpose of these techniques is to find or to define groups in the data-set.

The first step in the analysis of a multivariate data-set should always be the display, as in this way an indication can be obtained of what can be expected from further multivariate investigations. In this article it is the intention to introduce and to illustrate these methods with results obtained from a specific data-set. Besides the typical display methods, some supervised and most clustering techniques also permit visualization of the data. So the application of such techniques on the same data-set is also explained.

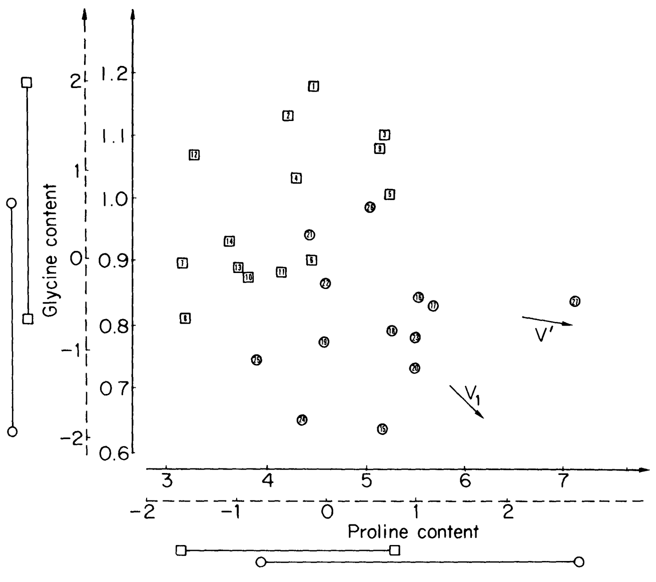

*Figure 1. Plot of the concentration of the proline content against the concentration of the glycine content in 14 Beaujolais wines (1–14) and 13 Côte du Rhone wines (15–27). The full lines are the axes of the plot of the unscaled data; the dotted lines are the plot of the scaled data. Where □——□ = range of the parameters in the Beaujolais wines; and ○——○ = range of the parameters in the Côte du Rhone wines.*

### Data-set

195 French wines were analysed for their amino-acid pattern. An appropriate sample of wine was boiled for 20 h in a nitrogen atmosphere with an excess of 6N HCl. After filtration and concentration in vacuum an adequate amount of the concentrate was injected into an amino-acid analyser (a Technicon NC-2P). The concentration of each of the 20 identifiable amino-acids was determined by the use of an external standard that contained each of the amino-acids in an amount comparable to that of the samples.

The wines that were analysed can be roughly divided into three categories: 110 Bourgogne wines, 13 Côtes du Rhone wines and 72 Bordeaux wines. In the first group a distinction can be made between 71 Beaujolais wines and 39 non-Beaujolais wines, including wines from the Macon and Cote d'Or regions. Table 1 gives the mean concentration and standard deviation of each of the 20 amino-acids in each of these four groups of wines.

*Table 1. Mean and standard deviation of the amino-acids (mmol/l) in each of the four groups of French wines.*

| | Bourgogne: Beaujolais ($N=71$) | Bourgogne: non-Beaujolais ($N=39$) | Côtes du Rhone ($N=13$) | Bordeaux ($N=72$) |
|---|---|---|---|---|
| ASPac | 0·616±0·200 | 0·876±0·219 | 0·537±0·130 | 0·462±0·114 |
| THR | 0·509±0·187 | 0·580±0·183 | 0·305±0·071 | 0·284±0·070 |
| SER | 0·534±0·175 | 0·700±0·183 | 0·408±0·077 | 0·392±0·086 |
| GLUac | 1·355±0·039 | 1·860±0·586 | 0·965±0·199 | 0·711±0·177 |
| PRO | 3·635±0·731 | 5·011±0·705 | 5·165±0·798 | 9·303±2·026 |
| GLY | 0·920±0·230 | 1·190±0·228 | 0·805±0·103 | 0·715±0·139 |
| ALA | 1·334±0·547 | 1·961±0·722 | 0·806±0·186 | 0·783±0·206 |
| CYS | 0·165±0·072 | 0·217±0·046 | 0·178±0·062 | 0·131±0·032 |
| VAL | 0·364±0·130 | 0·510±0·139 | 0·276±0·075 | 0·288±0·072 |
| MET | 0·044±0·023 | 0·081±0·029 | 0·036±0·026 | 0·040±0·017 |
| ILE | 0·210±0·082 | 0·319±0·096 | 0·167±0·046 | 0·172±0·046 |
| LEU | 0·262±0·099 | 0·395±0·122 | 0·231±0·072 | 0·254±0·070 |
| TYR | 0·109±0·085 | 0·177±0·073 | 0·067±0·079 | 0·089±0·068 |
| PHE | 0·128±0·102 | 0·214±0·078 | 0·036±0·070 | 0·076±0·074 |
| GABA | 0·962±0·461 | 1·160±0·534 | 0·281±0·112 | 0·220±0·074 |
| HIS | 0·106±0·067 | 0·135±0·055 | 0·100±0·022 | 0·068±0·034 |
| ORN | 0·176±0·236 | 0·414±0·495 | 0·095±0·053 | 0·082±0·075 |
| LYS | 0·265±0·077 | 0·400±0·106 | 0·266±0·062 | 0·251±0·059 |
| ETH | 0·048±0·008 | 0·058±0·011 | 0·038±0·012 | 0·038±0·009 |
| ARG | 0·598±0·601 | 1·222±1·408 | 0·364±0·158 | 0·092±0·107 |

### Computer programs

The computer package 'ARTHUR' [11] was used to obtain principal component plots and non-linear maps of the data-set, the 'BMDP' package [12] was used for the application of LDA. From the 'CLUSTAN' package [13] the routines 'Hierarchy', 'Plink' and 'Tree' were used for the application of the clustering technique and for the representation of the corresponding dendrogram.

### Pre-treatment of the data

Before the application of the multivariate techniques, the data were transformed in order to make the range between the different variables comparable. A z transformation, or autoscaling, was applied: each variable is given variance one and mean zero. This means that from each variable the average of that variable over the entire data-set is subtracted and the result is divided by the standard deviation of that variable over the data-set. It will be demonstrated that scaling can have a great influence on the display obtained.

### Methods

### *Pure display methods*

A distinction can be made between linear and non-linear methods. The most frequently used linear display method is principal component analysis (PCA). Starting from the original $N$ variables, $N$ new uncorrelated variables, called principal components or latent variables, are calculated. These latent variables are linear combinations of the original parameters and are defined so that the first PC explains the greatest part of the variance within the data-set, the second PC is orthogonal to the second and explains the great part of the residual variance and so on. Each PC $U_j$ ($j=1, N$) is given by the linear function:

$$U_j = a_{1j} X_1 + a_{2j} X_2 + \ldots + a_{nj} X_n. \qquad (1)$$

The coefficients $a_{ij}$ (the loadings) give an indication of the importance of the original variable $i$ in the direction of $PC_j$. Instead of thinking of the objects as situated in the hyperspace with the position of each object given by the values of the $N$ original parameters, the objects can be thought now to be situated in a hyperspace the dimensions of which are given by the latent variables. This coincides with the rotation of the original space. The position of the samples in this rotated sphere are then given by the scores of each object on each PC. These scores are calculated by filling in the values of the parameters $X_i$ in equation (1).

As the first few PCs explain the greatest part of the variance within the data, the samples can be represented in a reduced space defined by these first few PCs only. The percentage of the variance explained by each PC can be calculated and gives an idea of how much of the information in the data-set is represented in the display.

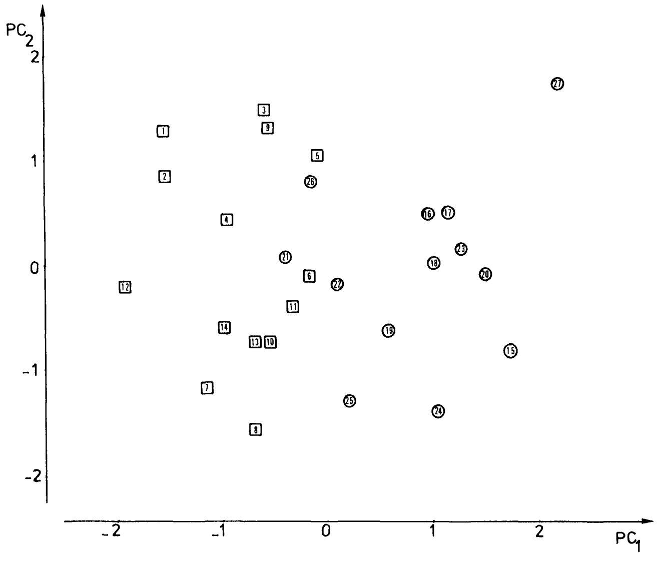

*Figure 2. Principal component plot of the scaled data in figure 1. Number of PCs used = 2.*

A PC analysis was performed on the scaled data-set plotted in figure 1. As in this data-set each sample is characterized by two parameters, two PCs can be computed. The vector $V_1$ in figure 1 gives the direction of the first PC. The second PC will be orthogonal to the first. The PCs are given by the following equations:

$$U_1 = 0{\cdot}7071\ X_1 - 0{\cdot}7071\ X_2$$

$$U_2 = 0{\cdot}7071\ X_1 + 0{\cdot}7071\ X_2.$$

Instead of representing the samples in the original pattern space, as was done in figure 1, they can also be represented in a rotated space—the co-ordinates of which coincide with the direction of the PCs (see figure 2). The position of, for instance, sample 1, which has in the plot of the scaled data the co-ordinates (−0·158, 1·997) is computed in the following way:

$$U_{1,1} = 0{\cdot}7071\ (-0{\cdot}158) - 0{\cdot}7071\ 1{\cdot}997 = -1{\cdot}524$$

$$U_{2,1} = 09{\cdot}7071\ (-0{\cdot}158) + 0{\cdot}7071\ 1{\cdot}997 = 1{\cdot}300.$$

In order to represent the data in a reduced space, i.e. in a one-dimensional space in this example (on one axis), the data points can be projected on the first PC: figure 3 shows such a display. As the fraction of the total variance explained by the first PC amounts to 57·8%, the display obtained in the reduced space gives an idea of the situation of the samples in the original space. The loadings of both parameters on the first PC are equal, which means that the variance represented in the direction of this PC is defined by both variables to the same extent. In fact, when PCA is performed on a two-dimensional data-set with autoscaled variables, the absolute value of the loadings on both PCs will always be equal to each other. In the case that more than two variables are included in the data-set, the loadings cannot be predicted *a priori*; the loadings of the variables on the important PC give an indication of the relations between the parameters themselves. Indeed, when two variables are strongly correlated the variance of these two variables over all the objects will vary in a similar way. In the case that a great part of the variance of one of these variables is explained, by for instance, PC 1, resulting in a high loading on this PC, a significant part of the variance within the other variable will also be explained by the same PC, so that the loading of this second variable will also be high on this same PC. Variables that have high loadings on the first few PCs will be strongly related to each other.

If PCA is performed on the unscaled data, the first PC explains 97·5% of the variance and the second 2·5%. This first PC is given by the equation:

$$U_1 = 0{\cdot}9997\ X_1 - 0{\cdot}0251\ X_2.$$

Clearly the direction of this PC is given almost solely by proline—the loading of this variable is much greater than that of glycine. Consequently, the information given by this PC (see figure 4) is almost the same as that given by proline itself. This is to be expected as the variance of proline in the samples (0·8906) is much greater than that of glycine (0·1442).

If the only purpose of displaying the data is to obtain a visualization of the data-set without interest in the relations between the parameters used to characterize the samples, non-linear display methods can be used as well. These methods produce displays in which the reduced dimensions are non-linear combinations of the original variables. The displays one obtains are called maps. Non-linear mapping (NLM) is one of the most frequently used methods: it represents the data in a reduced dimension according to the criterion that the distances between the samples in the reduced space approach the distances between the samples in the original space. Kowalski and Bender [14] give the following comparison in order to explain the method. Suppose that each of the samples in the original hyperspace is connected with every other sample by tensionless springs. The total energy of the springs is zero when considered in the original space. Pressing the samples together in order to obtain a mapping into, for instance, two dimesions gives rise to a tension on each of the springs. NLM aims to give a representation of the data so that the sum of the tension on all the springs is minimal.

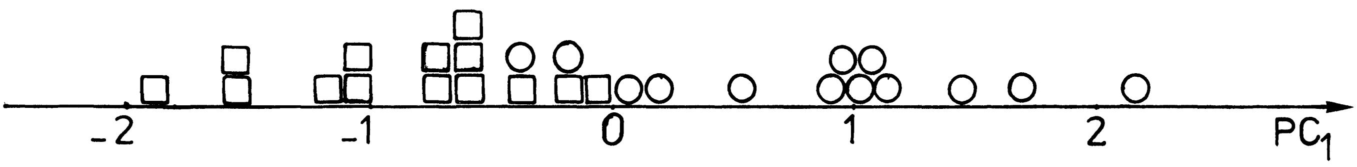

*Figure 3. Principal component plot of the scaled data in figure 1. Number of PCs used = 1.*

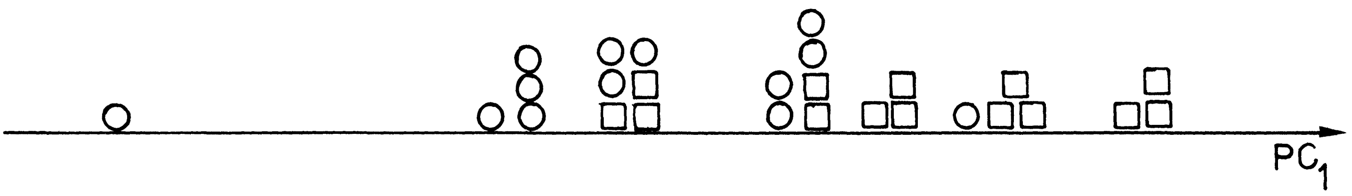

*Figure 4. Principal component plot of the unscaled data in figure 1. Number of PCs used = 1.*

*Application to the wine data*

Figure 5 represents the display obtained by the application of PCA on the complete scaled data-set. The percentage of the variance explained by PC 1 and PC 2 amounts respectively to 67·7% and 8·2%. Clearly, a great part of the variance within the data has already been explained by one component. From the display it becomes obvious that wines can be fairly well differentiated on the basis of their amino-acid spectrum. The Bordeaux wines, particularly, form a compact cluster in the plot. The differences between the two subgroups in the Bourgogne wines is less explicit, but a certain degree of dissimilarity can still be seen.

Table 2 gives the loadings of the amino-acids on the two first PCs. The loadings on PC 1 have the same magnitude for all the parameters (except for proline but this has practically no importance in the first direction). This means that this PC represents mainly the difference in the concentration of the amino-acids in the samples. As the Bordeaux and the Côtes du Rhone wines all have a low score on this component, it can be concluded that these groups of wines are characterized by a lower total amino-acid content as compared to the Bourgogne wines. The direction of the second PC is largely due to the proline content. The discrimination that occurs in this direction between Beaujolais wines and the other groups is mainly due to the greater concentration of proline in Beaujolais.

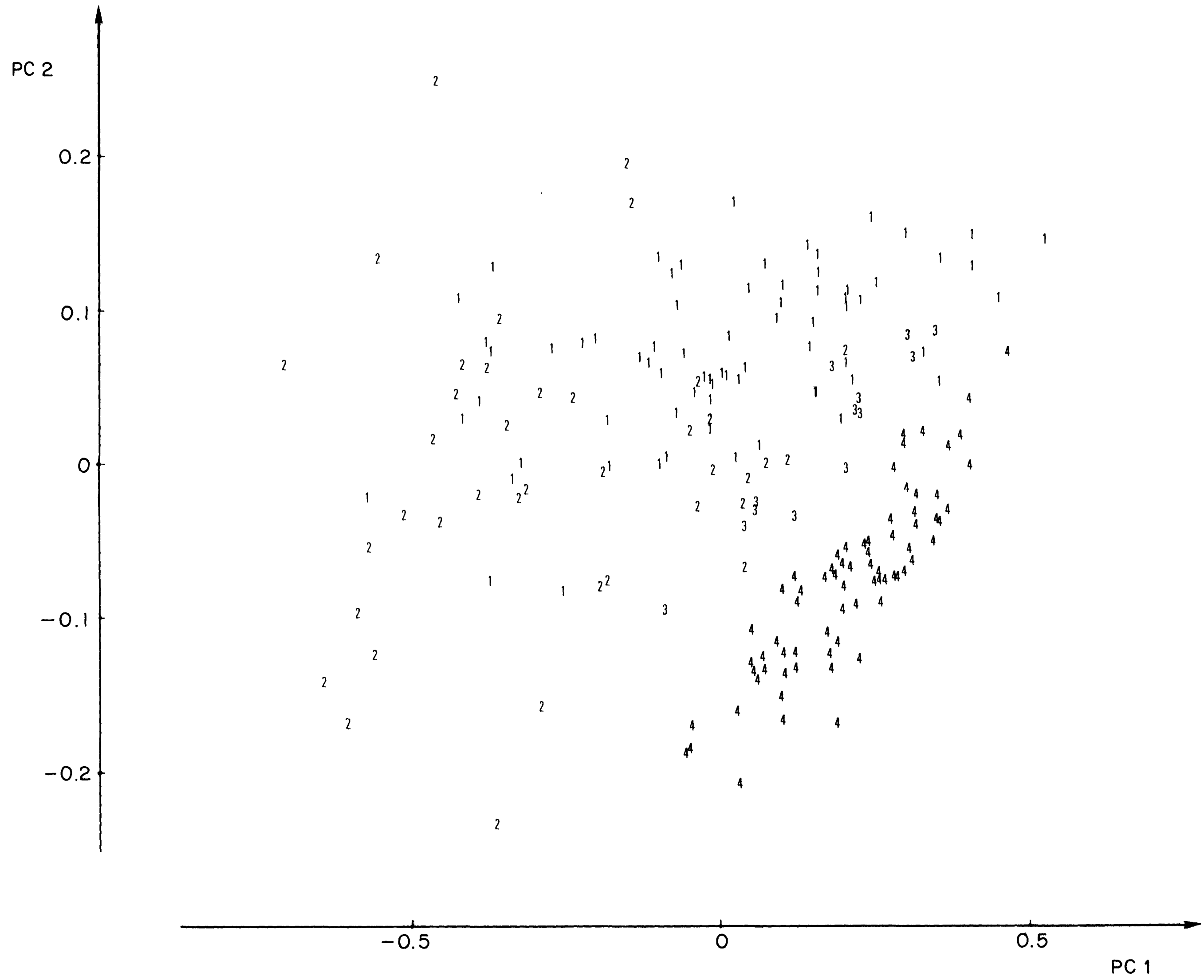

*Figure 5. Principal component plot of 195 French wines characterized by the concentration of 20 amino-acids (scaled data). Where 1 = Beaujolais wines; 2 = non-Beaujolais Bourgogne wines; 3 = Côte du Rhone wines; and 4 = Bordeaux wines.*

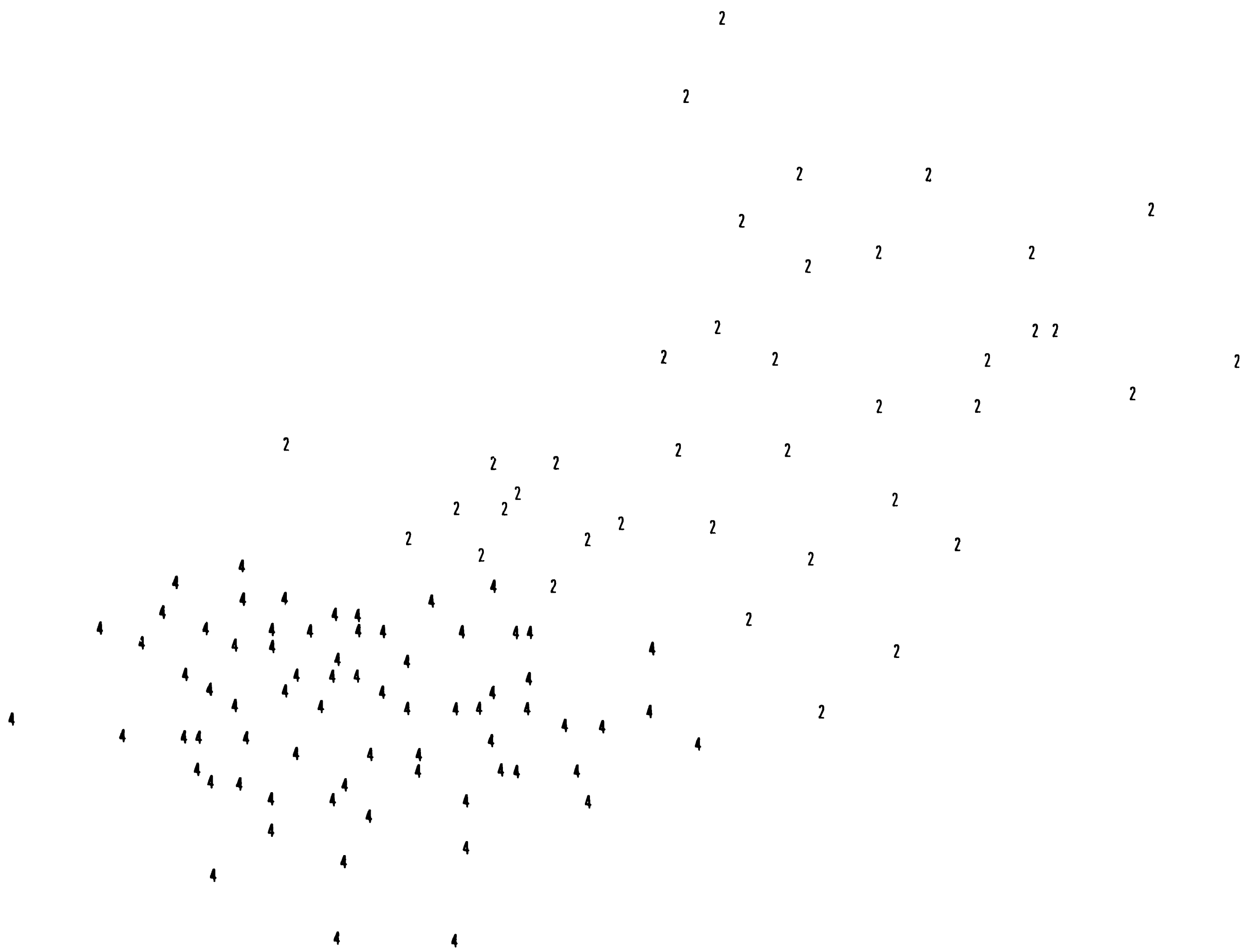

*Figure 6. Non-linear map of 72 Bordeaux wines (4) and 39 non-Beaujolais Bourgogne wines (2).*

As mentioned above, the loadings on the most important PCs may give an indication of the relationship between the parameters themselves. In the example, only the first PC is really important. The amino-acids Asp-ac, Thr, Ser, Glu-ac, Gly, Ala, Val, Met, Ile, Leu, Phe and Lys have almost the same weight on this PC; consequently, these parameters must be correlated to a high degree. This conclusion was confirmed by the application of the routine Pearson available in the SPSS package [15]. The correlation between each of the amino-acids mentioned ranges from 0·68 to 0·95. There is no significant correlation between proline and one of the other amino-acids.

The second display method applied was NLM. As the method is time-consuming, the NLM routine of the ARTHUR package was used to obtain maps for two groups of wine at a time. Figure 6 is the map of the Bordeaux and the non-Beaujolais Bourgogne wines; figure 7 is the map of the Bourgogne wines. Again, it can be concluded that a differentiation of wines must be possible on the basis of the parameters used in the study. In order to compare the representation obtained with NLM and PCA, PCA was applied to the same combinations of two groups of wines. The pictures obtained with NLM are similar to the PC plots. As the optimization procedure to obtain an NLM map is rather tedious, and no supplementary information is obtained about the parameters themselves (i.e. it cannot be easily concluded which parameters are responsible for the differentiation between the groups), PCA is the preferred display method.

*Table 2. Loadings of the parameters on the two first principal components.*

| | PC 1 | PC 2 |
|---|---|---|
| ASPac | –0·2616 | –0·0660 |
| THR | –0·2484 | 0·1029 |
| SER | –0·2627 | –0·2513 |
| GLU | –0·2510 | 0·2164 |
| PRO | 0·0848 | –0·5838 |
| GLY | –0·2558 | 0·0119 |
| ALA | –0·2539 | 0·1317 |
| CYS | –0·2048 | –0·0500 |
| VAL | –0·2643 | –0·1062 |
| MET | –0·2402 | –0·2473 |
| ILE | –0·2605 | –0·1505 |
| LEU | –0·2401 | –0·3042 |
| TYR | –0·1845 | –0·1652 |
| PHE | –0·2350 | –0·1547 |
| GABA | –0·2187 | 0·3465 |
| HIS | –0·1985 | 0·1197 |
| ORN | –0·1216 | 0·1664 |
| LYS | –0·2354 | –0·2610 |
| ETH | –0·1706 | 0·1561 |
| ARG | –0·1732 | 0·2901 |

### *Supervised methods that can be used for display*

As mentioned above, supervised pattern-recognition techniques are methods that deal with the problem of the classification of

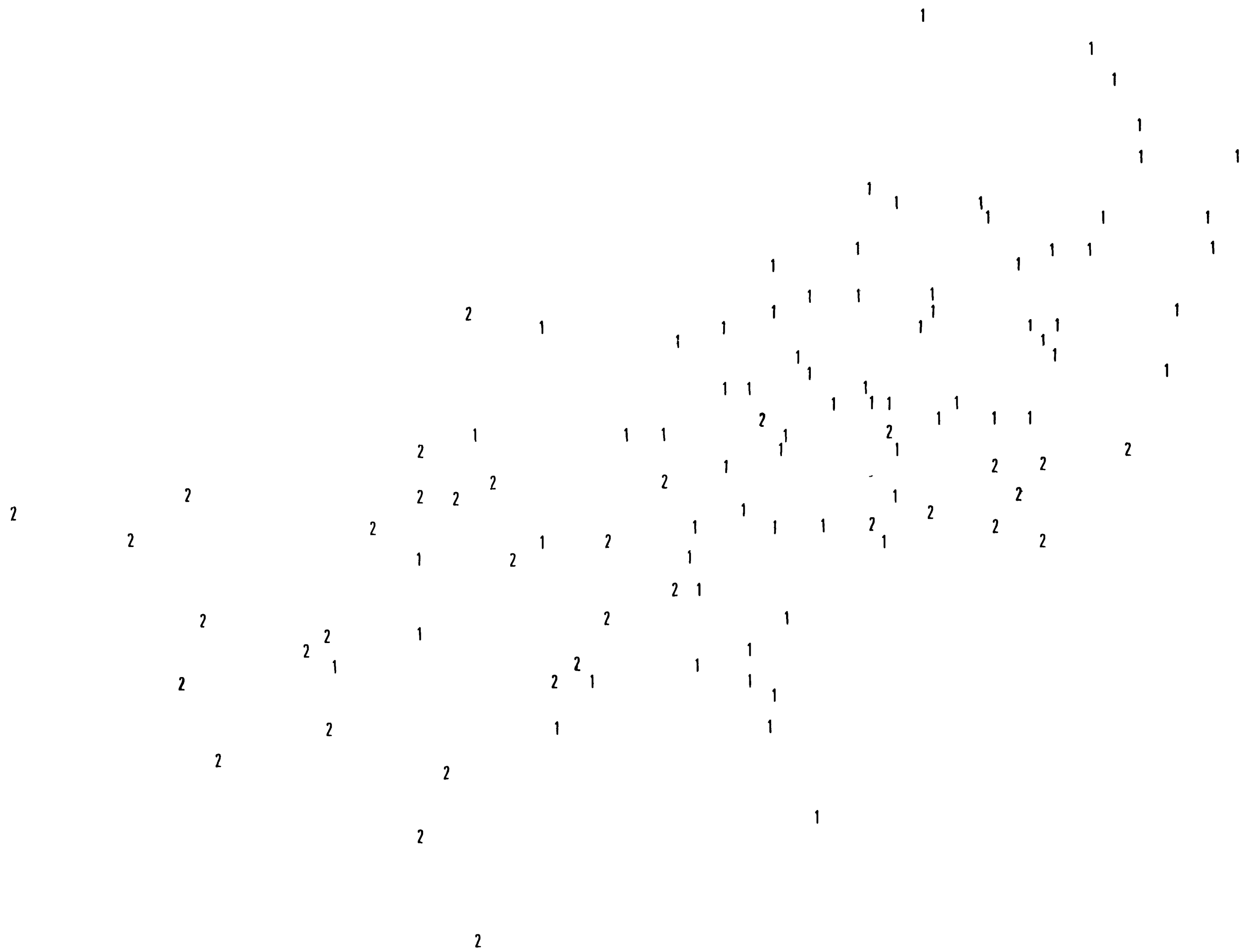

*Figure 7. Non-linear map of 110 Bourgogne wines. Where 1 = Beaujolais wines; and 2 = non-Beaujolais Bourgogne wines.*

samples or objects into a group. The general procedure carried out in a supervised situation is as follows. First, with a data-set consisting of objects with known classification, a classification or decision rule is developed that separates the learning classes in an optimal way—these decision rules can be used for the classification of new samples. The computation of the decision rules corresponds with the division of the original hyperspace in as many regions as there are learning classes in the training set, each region corresponding to one class. New objects are classified according to their position with respect to the boundaries between the classes. The data-set collected on the wines can obviously be investigated with supervised techniques as the samples can *a priori* be divided into several groups, i.e. into groups of wines from the same region. Some supervised techniques, such as linear discriminant analysis (LDA), also give a display. In LDA, the classification functions are developed in a reduced pattern space; as in PCA, each of the directions of this space is obtained by a linear combination of the original variables. These new variables are called discriminant functions or canonical variates. The criterion used to define the discriminant function is the maximization of the ratio of the between-group variance to the within-group variance. The resulting discriminant functions are oriented in the direction which provides a maximum differentation between the classes. The dimension of the reduced pattern space is one less than the number of training classes. In the case of two groups, one discriminant function is obtained. Each of the discriminant functions is given by the equation:

$$\mathrm{DF}_i = a_{1j} X_1 + \ldots + a_{nj} X_n. \qquad (2)$$

The position of an object, $k$, in the reduced space is given by its score on each discriminant function:

$$ds_{ik} = a_{1j} X_{1k} + \ldots + a_{nj} X_{nk}. \qquad (3)$$

The display obtained with this technique visualizes the optimal discrimination between the classes. The vector $V'$ in figure 1 gives the direction of the discriminant function obtained when LDA was applied to the data-set represented in the figure. Figure 8 gives the display of the data in the reduced one-dimensional space.

*Application to the wine data*

With LDA, a display of the data-set is obtained when the samples are plotted in the space defined by the discriminant functions. When a discrimination is attempted between three groups, two discriminant functions are calculated and the optimum discrimination can be represented in a two-dimensional plot. If the differentiation between, for instance, four groups is investigated, the reduced space is three-dimensional and so visualization becomes more difficult. Therefore LDA was applied to only three groups at a time. Figure 9 gives the plot obtained for the two subgroups in the Bourgogne wines and the Bordeaux wines. The plot shows that these three groups can be discriminated very well on the basis of their amino-acid pattern, even the two subgroups in the Bourgogne wines can be separated in this plot.

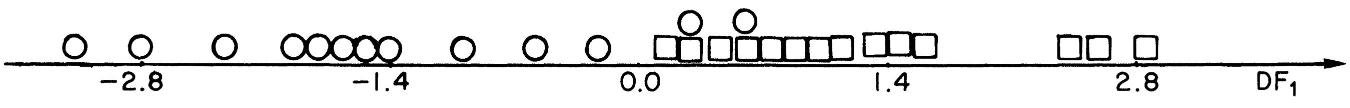

*Figure 8. LDA plot of the data in figure 1.*

*Table 3. Coefficients of the values of the variables on the canonical variates. The parameters are given in order of inclusion.*

| | | |
|---|---|---|
| PRO | −2·0961 | −0·0124 |
| THR | 0·7964 | 1·7510 |
| ILE | −1·3110 | −0·9765 |
| ETH | 0·4535 | −0·3658 |
| CYS | 0·5201 | 0·0569 |
| ARG | −0·3250 | −0·7462 |
| GABA | −0·6048 | 0·5115 |
| ORN | −0·0360 | −0·6280 |
| HIS | 0·0014 | 0·7187 |
| ASPac | 0·4468 | −1·6433 |

The variables were introduced in the discriminant function one at a time. The selection criterion for the introduction of a new parameter is based on the discriminating power of that variable, i.e. the degree to which a newly introduced variable increases the discrimination between the groups. Variables are included in the discriminant function until the further inclusion of one of the remaining parameters does not significantly improve the discrimination. The coefficients of the canonical variates obtained in this application are given in table 3. Only the amino-acids Asp-ac, Thr, Pro, Cys, Ileu, Gaba, His, Orn, Eth, and Arg were included. It is this combination of amino-acids that gives the best discrimination between the three groups of wines under investigation.

### *Clustering techniques*

The aim of multivariate techniques belonging to this group is mainly to find groups of similar samples in the data-set and to display these in a so-called dendrogram. The first step in clustering is the determination of the similarity between the different objects to be clustered. One of the possible criteria is the distance between the samples in the original hyperspace. Consider, for instance, four objects (A, B, C, D) each measured for three different variables (X, Y, Z):

| | X | Y | Z |
|---|---|---|---|
| A | 0·1 | 2·1 | 1·5 |
| B | 0·2 | 1·9 | 1·4 |
| C | 0·2 | 1·8 | 0·2 |
| D | 1·0 | 0·4 | 0·2 |

As samples A and B have very similar results for each of the three parameters, they must be similar. Sample D, on the other hand, differs as much from A as from B. C is similar to A and B in terms of X and Y, but differs from both in the value of the third parameter. Considering the situation of the data in the pattern space (figure 10), it is evident that the more similar objects are the closer they are situated to each other: A and B are very near to each other, D is situated in a totally different part of the pattern space. A frequently used measure for the distance between objects in the pattern space is the Euclidian distance. The distance, i.e. the similarity, between two samples A and B is then computed by the formula:

$$d_{AB}=\left\{\sum_{i=1}^{i=N}(X_{ia}-X_{ib})^2\right\}^{1/2} \quad (4)$$

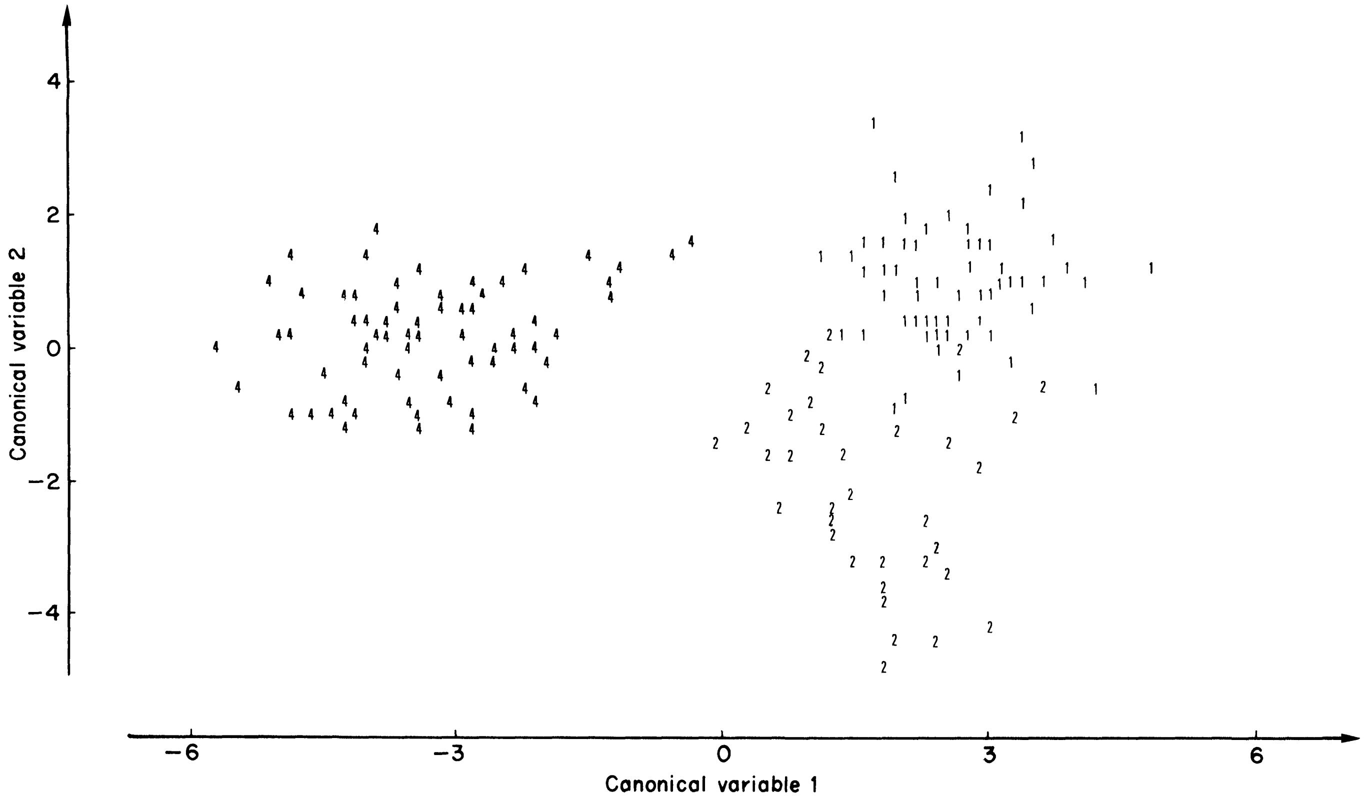

*Figure 9. LDA plot of the Bordeaux wines (4), the Beaujolais wines (1) and the non-Beaujolais Bourgogne wines (2).*

or:

$$d_{AB}=[(0{\cdot}2-0{\cdot}1)^2+(1{\cdot}9-2{\cdot}1)^2+(1{\cdot}4-1{\cdot}5)^2]^{0{\cdot}5}=0{\cdot}24.$$

The distances must be computed for each pair of objects and the results can be represented in a similarity matrix:

| | A | B | C | D |
|---|---|---|---|---|
| A | 1·00 | 0·24 | 2·34 | 2·32 |
| B | 0·24 | 1·00 | 1·20 | 2·08 |
| C | 1·34 | 1·20 | 1·00 | 1·61 |
| D | 2·32 | 2·08 | 1·61 | 1·00 |

The second step in the clustering procedure is the search for similar groups of objects. Many clustering methods exist, differing in the criteria used to decide which belong to the same group. One of the possible procedures is the following. In the first instance, all the objects are considered separately. Then the two most similar objects (A and B) are to form a cluster A*. The similarity between this cluster and the remaining objects is now represented by one value, for instance the mean similarity between the objects of the cluster and the other objects:

| | A* | C | D |
|---|---|---|---|
| A* | 1·00 | 1·27 | 2·20 |
| C | 1·27 | 1·00 | 1·61 |
| D | 2·20 | 1·61 | 1·00 |

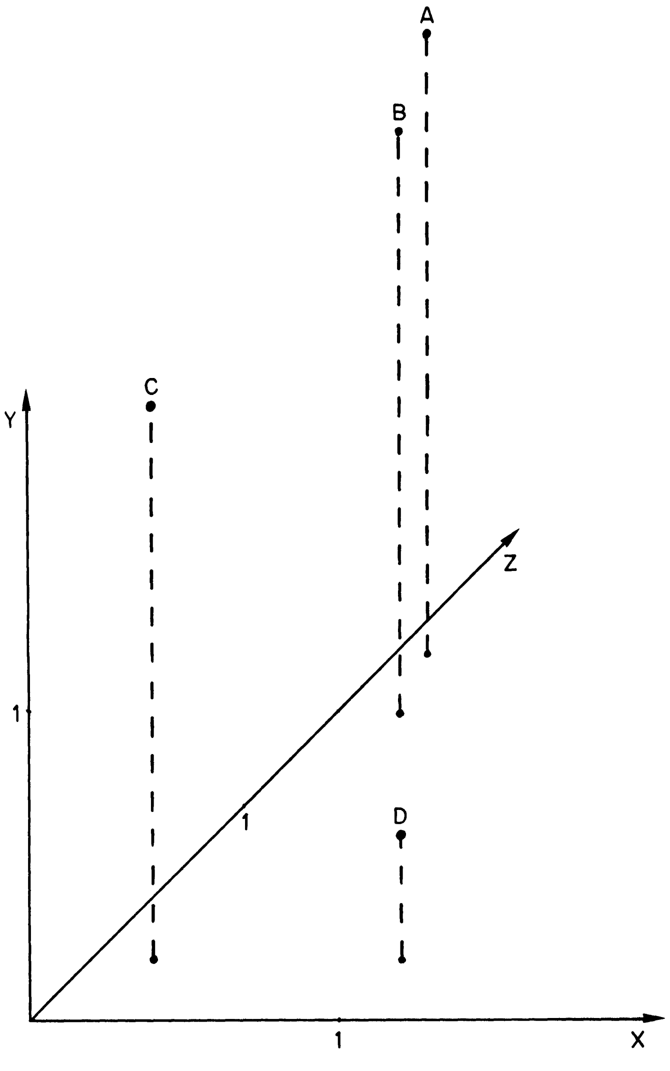

*Figure 10. Representation of the situation of four objects (each measured for three parameters) in the pattern space.*

Again, the two most similar objects or clusters, A* and C, are joined and represented as one object:

| | A* | D |
|---|---|---|
| A* | 1·00 | 1·91 |
| D | 1·91 | 1·00 |

This procedure continues until only one cluster containing all the objects remains. As the levels of clusters obtained are such that smaller clusters are always included into larger ones, methods based on this principle are called agglomerative hierarchical methods. The relations between the several levels of clustering can be represented in a dendrogram:

```
A     B     C     D
I _ _ I     I     I
            I     I
   I        I     I
   I _ _ _  I     I
                  I
   I              I
   I              I
   I              I
   I _ _ _ _ _ _  I
```

A dendrogram visualizes the relations between the different objects of the data-set: the total height of the links are a measure of the distances between the objects. The algorithm used to join the clusters is called the average linkage method, and the method used for clustering the data of figure 1 and the wine data is known as Ward's method, or the error sums of squares method. The clustering obtained with this method is generally comparable to that obtained with the average linkage algorithm. More information on the different clustering algorithms can be found in a recent book by Massart and Kaufman [16].

Figure 11 is the dendrogram of the scaled data of figure 1. As the objects can be divided into two classes, the highest link in this dendrogram can be cut to obtain two clusters and to see whether each of these contain mainly objects of a same class. Applying this criterion, a cluster is obtained which consists only of Côte du Rhone wines, while the second contains all the Beaujolais wines and three Côte Rhone wines. Obviously, the dendrogram visualizes the degree of discrimination between the groups of wines.

The application of Ward's method to the wine data results in the separation of the objects into two very distinct groups (see figure 12): group A is compact and consists mainly of Bordeaux wines; group B, which is more heterogeneous, can be divided into three subgroups: B1 and B3 consist of Bourgogne wines and B2 joins wines from Bordeaux and from Bourgogne. The Côte du Rhone wines appear in all of the clusters.

### Conclusion

The object of this paper is to show that display and related pattern-recognition techniques permit the investigation of multivariate data-sets. Wine analysis is considered only as an example and certain aspects are treated more fully by Ooghe and De Waele [10]. Clearly, the applications are not confined to wine or food analysis. Applications in, for example, archeometry [17], meteoritics [18], microbiology [19], medical diagnosis [20], investigations of structure activity relations [21], and environmental problems [22] have also been reported. Pattern recognition is a natural addition to automated analysis: eventually there will be instruments which can analyse samples, determine the pattern to which a sample corresponds, and suggest its geographical origin.

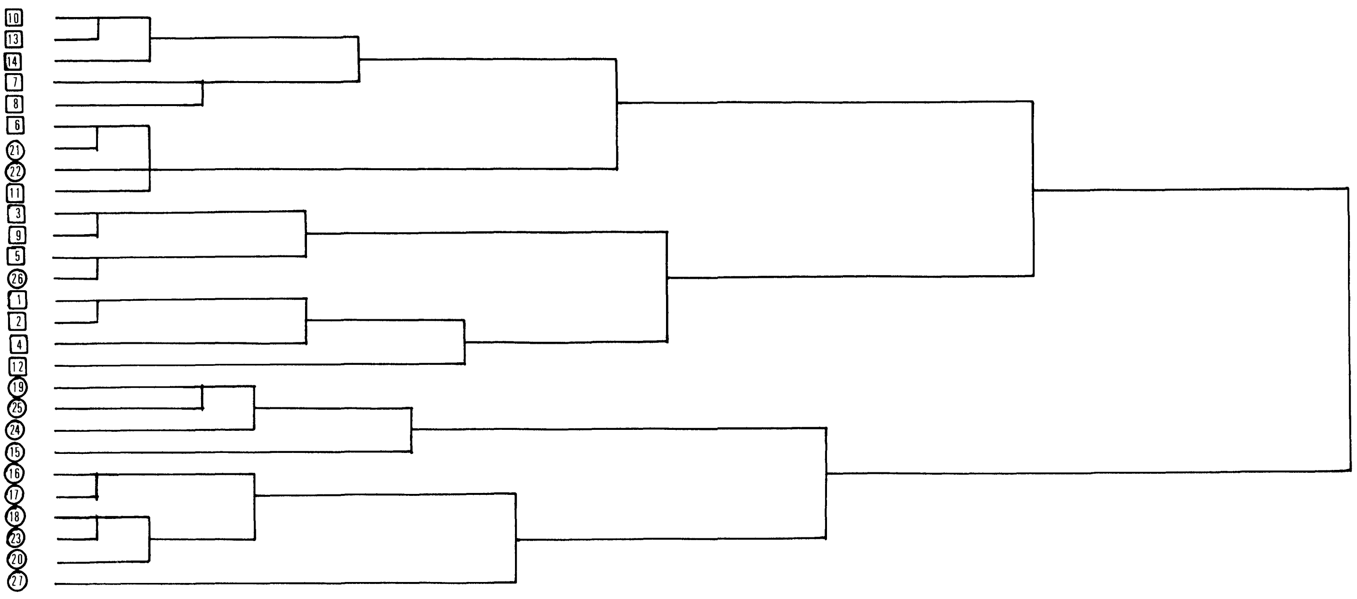

*Figure 11. Dendrogram of the scaled objects in figure 1 (obtained by using the Ward's method).*

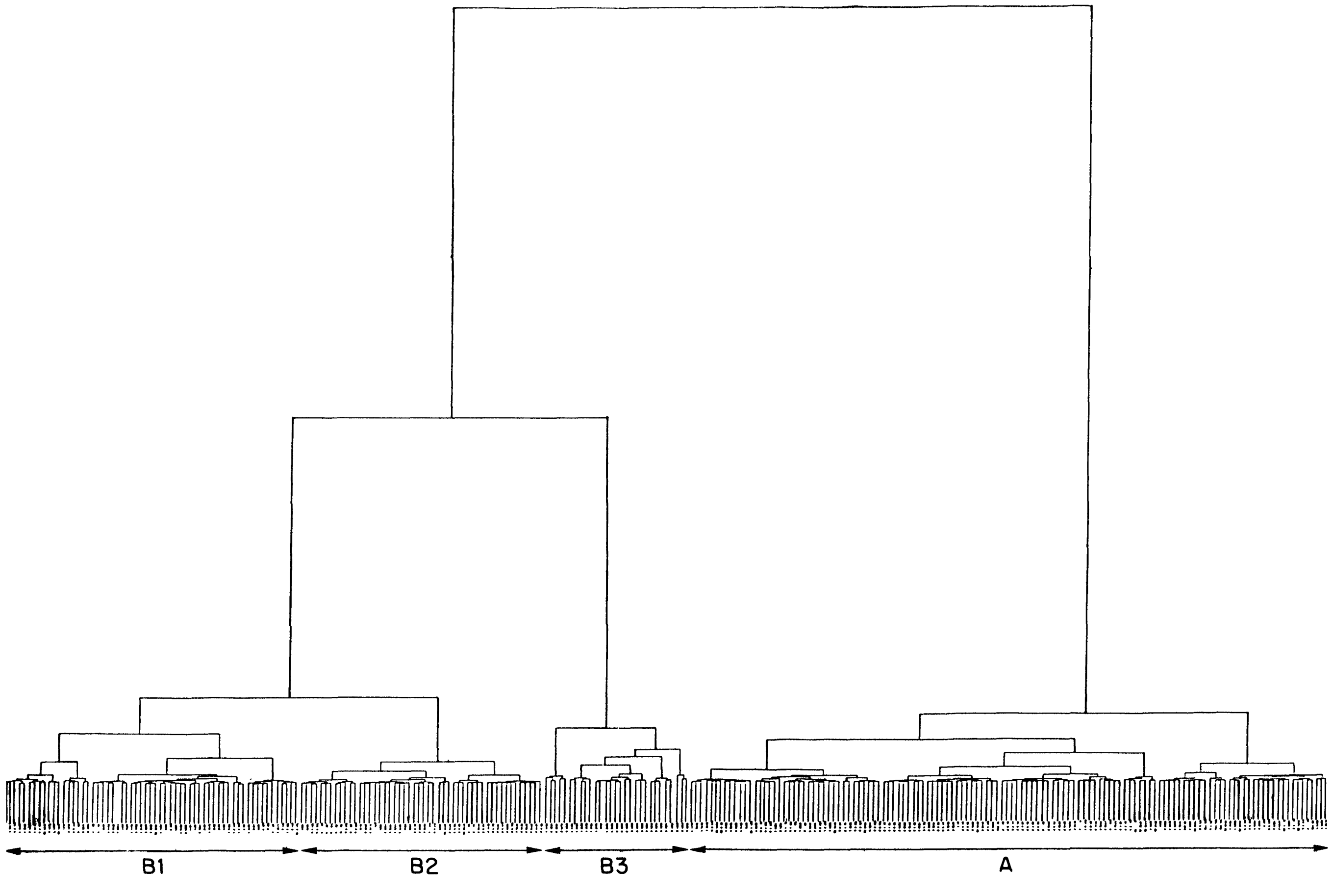

*Figure 12. Dendrogram of the wine data (obtained by using Ward's method).*

### References

1. Moret, I., Scarponi, G., Capodaglio, G., Zanin, S. Camaiani, G. and Toniolo, A., *American Journal of Ecology and Viticulture*, **32,** 3 (1980), 245.
2. Scarponi, G., Moret, I. and Capodaglio, G., *G. Riv. Vitic. Enol.*, **34,** 6 (1981), 254.
3. Scarponi, G., Moret, I., Capodaglio, G. and Cescon, P., *Journal of Agriculture and Food Chemistry*, **30,** 6 (1982), 1135.
4. Kwan, W. O., Kowalski, B. R. and Skogerboe, R. K., *Journal of Agriculture of Food Chemistry*, **27,** 6 (1979), 1321.
5. Schreier, P., Drawert, F., Junker, A. and Reiner, L., *Mitteilungen Rebe und Wein Obstrau und Fruchteverwertung* (1976), 225.
6. Bielig, H. J. and Askar, A., *Chemie, Mikrobiologie, Technologie der Lebensmittel*, **1** (1972), 183.
7. Habegger, M. and Sulser, H., *Lebensmittel-Wissenschaft und Technologie*, **7,** 3 (1974), 11.
8. Ooghe, W., *Voedingsmiddelentechnologie*, **13,** 15 (1980).
9. Gilbert, J., Shepherd, M. J., Wallwork, M. A. and Harris, R. G., *Journal of Agricultural Research*, **20,** 2 (1981), 125.

10. Ooghe, W. and de Waele, A., *Ann. Fals. Exp. Chim.*, **74** (1981), 381.
11. Duewer, D. L., Koskinen, J. R. and Kowalski, B. R., ARTHUR (available from B. R. Kowalski).
12. Dixon, W. J., *BMDP Biomedical Computer Programs* (University of California Press, Berkeley, 1975).
13. Wishart, D., *Clustan Users Guide* (The Clustan Project, University College, 19 Gordon Street, London, 1975).
14. Kowalski, B. R. and Bender, C. F., *Journal of the American Chemical Society*, **95**, 3 (1973), 686.
15. Nie, N. N., Hull, C. H., Jenkins, J. G., Steinbrenner, K. and Bent, D., *Statistical Package for the Social Sciences (SPSS)*, (McGraw Hill, New York 1975).
16. Massart, D. L. and Kaufman, L., *The Interpretation of Analytical Chemical Data by the Use of Cluster Analysis* (John Wiley, New York, in press).
17. Kowalski, B. R., Schatski, T. F. and Stross, F. H., *Analytical Chemistry*, **44** (1972), 2176.
18. Massart, D. L., Kaufman, L. and Esbensen, K. H., *Analytical Chemistry*, **54** (1982), 911.
19. Wieten, G., Haverkamp, J., Meuzelaar, H. C. C., Engel, H. W. B. and Berwald, L. G., *Journal of General Microbiology*, **122** (1981), 109.
20. Coomans, D., Masart, D. L. and Kaufman, L., *Analytica Chimica Acta*, **112** (1979), 97.
21. McGill, J. R. and Kowalski, B. R., *Analytical Chemistry*, **49** (1977), 596.
22. Hopke, P. H., Gladney, E. S., Gordon, G. E., Zoller, W. H. and Jones, A. G., *Atmospheric Environment*, **10** (1976), 1015.

### PARTICLE TECHNOLOGY SEMINAR

On 5 and 6 October 1983 Micromeritics are holding a two-day seminar in Atlanta, Georgia, USA (four others are being run in different locations during September 1983). Micromeritics' particle technology seminars were first introduced in 1982 and are a comprehensive overview for both new and experienced practitioners of particle technology science.

Six sessions provide a balance of theory and practice for: particle size analysis, surface area analysis, pore structure analysis, chemisorption analysis, density measurement, and zeta potential measurement. A particle technology introduction and applications overview are included. A course workbook and lunches are provided; the cost is $100/day or $150 for both days.

*Registration information from Carol Stancel, Micromeritics Instrument Corporation, 5680 Goshen Springs Road, Norcross, Atlanta, Georgia 30093, USA. Tel.: 404 448 8282.*

### WORKSHOP IN LIQUID SCINTILLATION COUNTING

*5–9 September 1983, Queen Elizabeth College, London*

This is the third workshop on liquid scintillation counting to be organized by the Radiochemical Methods Group of the Analytical Division, Royal Society of Chemistry. The course is intended to provide a sound introduction to the technique; lectures include

- 'Atoms, isotopes and radionuclides' (Dr G. Ayrey)
- 'The scintillation process' (Mr M. A. Crook)
- 'Instrumentation' (Dr A. Dyer)
- 'Standardization and efficiency determination' (Mr C. J. Roberts)
- 'Computing errors, statistics and computation' (Dr D. Bowyer)
- 'Experimental design' (Dr P. Stanley)
- 'Homogeneous sample preparation' (Professor D. M. Taylor)
- 'Heterogeneous sample preparation' (Dr D. Dyer)
- 'Safety both chemical and radiochemical' (Dr P. Stanley)
- 'Automation and computing' (Mr G. Sutton)
- 'Discussion of experimental results' (Dr D. Bowyer)
- 'Other and novel counting techniques' (Dr A. Ware)

There will also be practical work, discussion periods and a poster session.

Support in the form of modern equipment and expertise has been promised from: Amersham International, Beckmann RIIC Ltd, Fisons Scientific Apparatus, Koch-Light Laboratories Ltd, Kontron Ltd, Laboratory Impex Ltd, LKB Instruments Ltd, Nuclear Enterprises Ltd, Packard Instruments Ltd, Philips (Pye Unicam Ltd) and Rotheroe and Mitchel Ltd.

The fee for the course will be £280 and includes accommodation in the College together with all meals except dinner on one free evening.

*Further information may be obtained from Dr G. Ayrey, Director of the Isotope Unit, Queen Elizabeth College (University of London), Campden Hill, London W8 7AH.*

